# Prescription of Psychotropic Drugs for Chronic Insomnia in Primary Care

**DOI:** 10.1111/jsr.70334

**Published:** 2026-04-26

**Authors:** Jesús Escribá‐Alepuz, Lidia Herrero‐Huertas, María García‐Gil, Estefanía Estévez‐López, Francisco Javier Puertas, Manuel de Entrambasaguas

**Affiliations:** ^1^ Sleep Medicine Institute Valencia Spain; ^2^ Clinical Neurophysiology Unit Sagunto Health Department Sagunto (Valencia) Spain; ^3^ Health Psychology Department Miguel Hernandez University Elche (Alicante) Spain; ^4^ National Epidemiology Centre Carlos III Health Institute Madrid Spain; ^5^ Pharmacy Service‐Primary Care, Sagunto Health Department Sagunto (Valencia) Spain; ^6^ Ribera University Hospital—FISABIO Alzira (Valencia) Spain; ^7^ Faculty of Medicine Catholic University of Valencia Valencia Spain; ^8^ Hospital Clínic Universitari de València, INCLIVA Biomedical Research Institute Valencia Spain

**Keywords:** epidemiology, hypnotics, insomnia

## Abstract

Insomnia is the most prevalent sleep disorder in adults, representing a major public health problem. Healthcare management in a specific area first requires analysing its current practices and issues. We conducted a retrospective observational study of over 100,000 patients from two large primary care databases in the public healthcare Department of Sagunto (Valencia, Spain), from 2009 to 2017. Cases of insomnia were defined by ICD‐10 codes, and prescriptions of hypnotics were collected. The data analysis was performed on epidemiological aspects, comorbidity, diagnosis and treatment of insomnia. A significant increase in the incidence of insomnia occurred, rising from 58.9 per 1000 inhabitants (95% CI: 57.7–60.0) in 2009 to 91.4 per 1000 inhabitants (95% CI: 90.0–92.9) in 2017. A total of 12,404 prescriptions for hypnotics and sedatives were issued, including 39.8% for indications other than insomnia. Lorazepam was the most frequently prescribed hypnotic‐sedative (36%), followed by zolpidem (21.1%); 54% of insomnia cases revealed more prescription units than medical consultations, suggesting automatic renewals. Excessive prescription of hypnotic drugs for insomnia, along with a lack of follow‐up, were identified as significant issues.

## 1 Introduction

Insomnia represents a prevalent sleep disorder characterised by nocturnal sleep difficulties and daytime impairment, as defined by DSM‐5 and the International Classification of Sleep Disorders (ICSD) criteria (Roth 2007; Ferre‐Maso et al. 2020; Paul and Salas 2024; Hafner et al. 2023).

Prevalence estimates range from 4% to 48%, depending on the case definitions. Restricting criteria incorporating daytime consequences, the prevalence range from 4% to 6% globally versus 10% to 48% based solely on nocturnal complaints. In Spain, chronic insomnia affects 6.0%–14.8% of adults consulting primary care, with symptoms in up to 43.4% of the Spanish population (Ellis et al. 2023; Morin and Jarrin 2022; Torrens et al. 2019). Prevalence rates rise in older adults, women and night‐shift workers, who are often excluded from case definitions to refine estimates (de Entrambasaguas et al. 2023; Lukacisinova et al. 2024; Zuo et al. 2022).

Most authors agree that the first‐line treatment to restore natural sleep processes is non‐pharmacological measures, such as cognitive‐behavioural therapy for insomnia (CBTi) (Riemann et al. 2023). However, benzodiazepines (BZDs) and receptor agonists dominate initial prescriptions despite short‐term efficacy (≤ 4 weeks) yielding to long‐term disruptions with reduced slow‐wave and REM sleep, rebound insomnia, next‐day impairment and heightened falls in the elderly (Hafner et al. 2023; DeKosky and Williamson 2020; Torres‐Bondia et al. 2022).

ICSD‐3 classifies hypnotic‐reliant patients fearing medication cessation—absent core symptoms—as insomnia cases, even with BZDs' off‐label anxiety uses. Spain is one of the countries with the highest consumption of BZDs, according to the International Narcotics Control Board and the Spanish Medicines Agency. It is estimated that in 2020, almost 110 doses per day were consumed in Spain per 1000 inhabitants. Only Belgium (84 daily doses) and Portugal (80) come close to Spain's figures, which are far ahead of geographically close countries such as Germany (0.04 daily doses) (Vicente Sánchez et al. 2013; Agencia Española del Medicamento 2025). Understanding why this situation exists is vital to amend it (Roncero et al. 2025).

The objective of this study is to describe the incidence and pharmacological management of chronic insomnia by primary care physicians in a specific area of Spain, the Sagunto Health Department, as a starting point to guide rational use aligned with sleep medicine guidelines.

## 2 Methods

This is a retrospective observational study to analyse the population frequency of insomnia diagnosis, according to the ICD‐10 coding system, and its pharmacological management by primary care professionals in the Sagunto healthcare area (Valencia, Spain) over a 9‐year period (2009–2017).

### 2.1 Population and Data Sources

The Sagunto Health Department has more than 152,000 inhabitants and comprises 50 municipalities in the regions of Camp de Morvedre (Valencia) and Alto Palancia (Castellón), as well as the municipalities of Puçol, El Puig (l'Horta Nord) and Almenara, Xilxes and La Llosa (La Plana Baixa) (Generalitat Valenciana 2025).

The population evolution of the municipality of Sagunto was obtained from population data published by the National Institute of Statistics (INE) for individuals aged 15 and over (INE 2024), considering that the INE's 5‐year age groups are defined in increments of 5.

Sagunto is a mid‐sized municipality in the province of Valencia (≈71,000–72,000 inhabitants according to the latest National Statistical Institute figures), and the health department administratively covers a mix of urban and rural basic health zones and multiple primary‐care centres (including ‘consultorios’ and metropolitan health centres), which allowed sampling across the urban–rural spectrum while remaining within a single, coherent health‐service structure.

Data on patients and treatments come from the two most important databases in the regional health network: Abucasis and PROSIGA (SIA‐GAIA) (Jiménez Cantos 2005; Fundació Fisabio—Generalitat Valenciana, n.d.). To match diagnosed cases of insomnia with the specific pharmacological prescriptions for each case, a random code was used to link each patient to their diagnosis and treatments.

### 2.2 Study Variables

For case selection and incidence estimation, the following ICD‐10 codes were defined as insomnia: 780.52; 780.51; 307.41; 307.42; 307.49; 291.82; 292.85; 327.00; 327.01; 327.02; 327.09; V69.5; 046.72.

Drugs used with and without indication for insomnia were analysed according to ATC codes like N05CH01 (melatonin agonists); N05CF01 (zopiclone) and N05CF02 (zolpidem); N05CD01 (flurazepam); N05CD05 (triazolam); N05CD08 (midazolam); N05CD06 (lormetazepam); N05CD11 (loprazolam), N05BA06 (lorazepam), N05BA01 (diazepam), N05BA12 (alprazolam). Information was collected on dispensing dates, start and end of treatment, dosage, frequency, prescription and active ingredient.

Comorbidities related to insomnia during the study period were also collected, along with the date and diagnosis code.

### 2.3 Analysis

A descriptive analysis was performed by calculating the central tendency and dispersion parameters for quantitative variables and frequency tables and percentage distributions for qualitative variables. The frequency of insomnia diagnostic codes in the 2009–2017 period in the specified health area was analysed, as well as the frequency of prescription and use of psychotropic drugs in these patients (type, dose, indication, administration). To calculate the incidence, the population denominator for the corresponding year was used, based on INE data as of 1 January for individuals aged 15 and over.

Stata v18 (StataCorp, College Station, Texas, USA) was used for the analyses.

## 3 Results

Between 2009 and 2017, a total of 107,124 new cases of insomnia were diagnosed in primary care in the Sagunto area of Valencia, with an increasing trend in the number of new cases per year, from 9125 cases in 2009 to 13,724 in 2017 in the health area studied. In terms of incidence, diagnoses increased from 58.9‰ in 2009 to 91.4‰ in 2017 (Figure 1).

**FIGURE 1:**
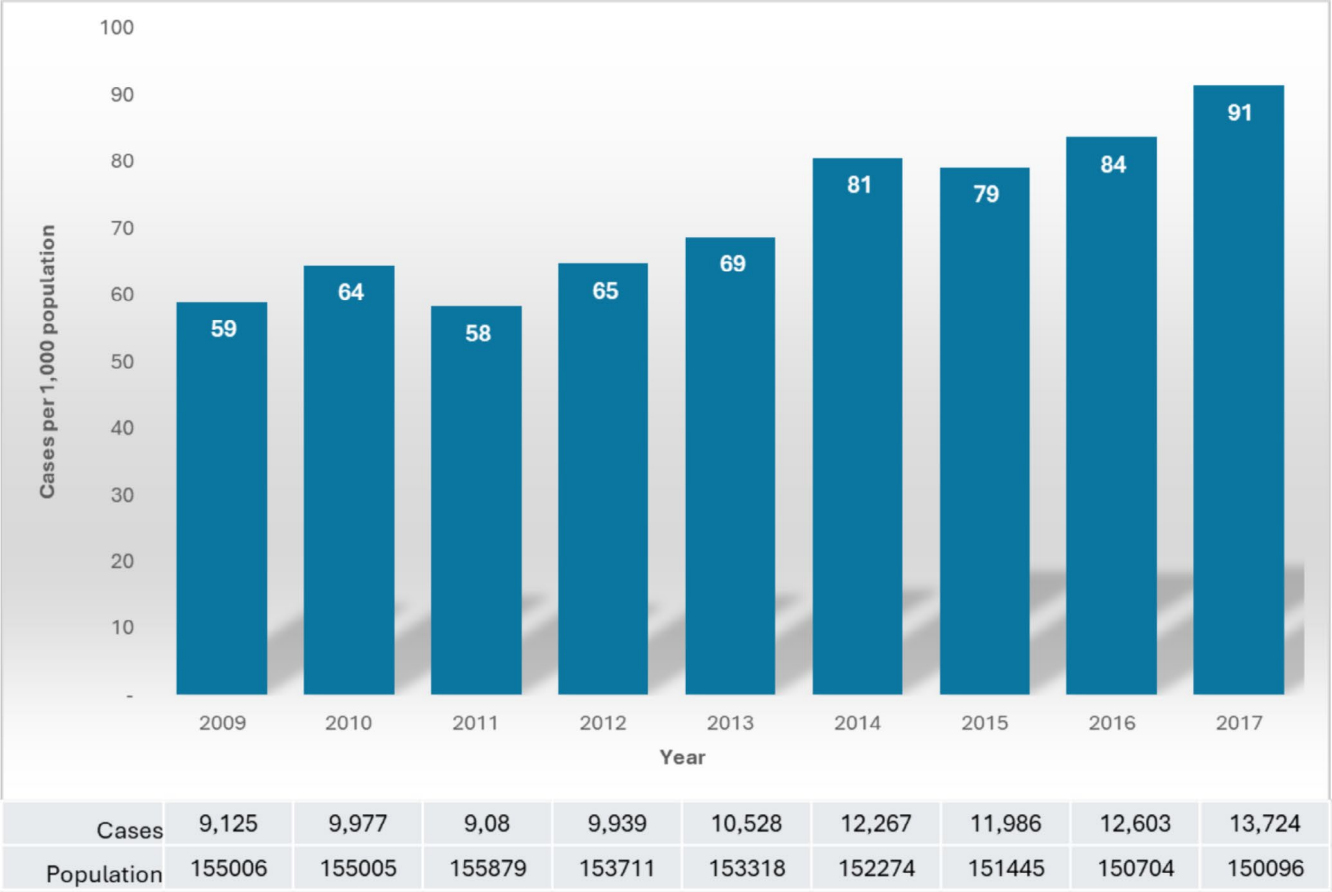
Yearly incidence of insomnia (×1000) in the Sagunto Health Department (Valencia, Spain, 2009–2017). *Source:* Primary care data from the Minimum Data Set (CMBD) from the Valencian Community, and the National Statistics Institute (INE).

The incidence of insomnia among people over 65 is much higher than among those under this age. In addition, there is a clear increase over time among older people, which is not as evident among those under this age. The same is true for women, with a higher prevalence of insomnia diagnosis than men (Figure 2).

**FIGURE 2:**
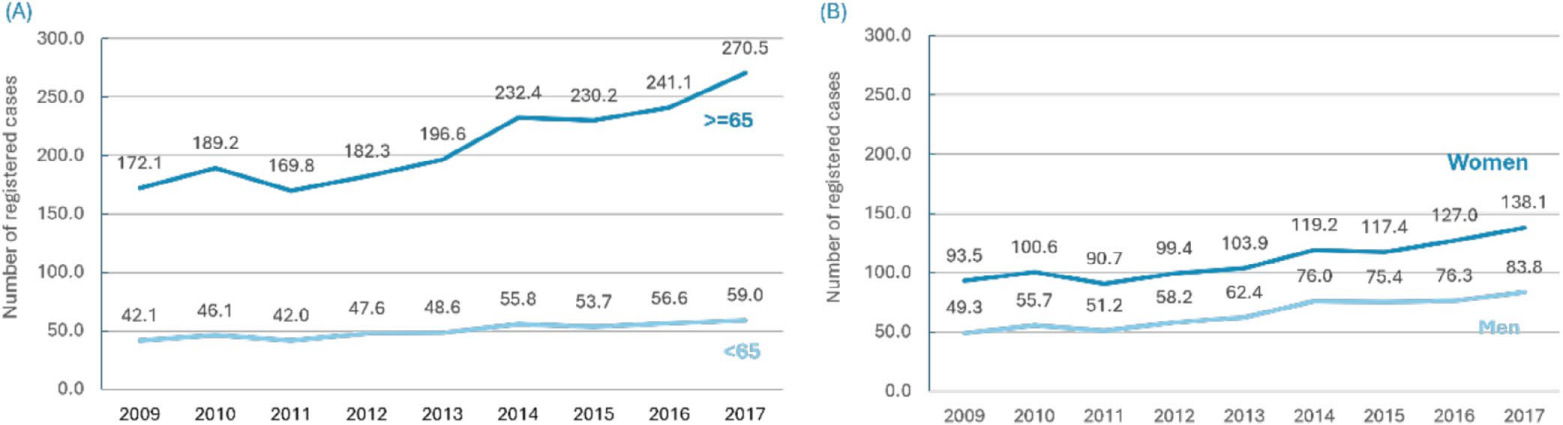
Yearly incidence of insomnia (×1000) in the Sagunto Health Department (Valencia, Spain, 2009–2017), by age over 65 (A) and sex (B). *Source:* Primary care data from the Minimum Data Set (CMBD) from the Valencian Community, and the National Statistics Institute (INE).

During the study period, a total of 14,923 prescriptions for hypnotics were recorded, of which 12,404 (83%) were associated with a diagnosis of insomnia. Insomnia was listed as the main indication in 60% of cases.

Lorazepam was the most commonly used hypnotic, accounting for 36% of all prescriptions, followed by zolpidem (21.1%), lormetazepam (14.3%) and diazepam (11.2%). Around 13% of prescriptions included other compounds or combinations of the above. Alprazolam was the least frequently prescribed compound (4.5%) (Figure 3).

**FIGURE 3:**
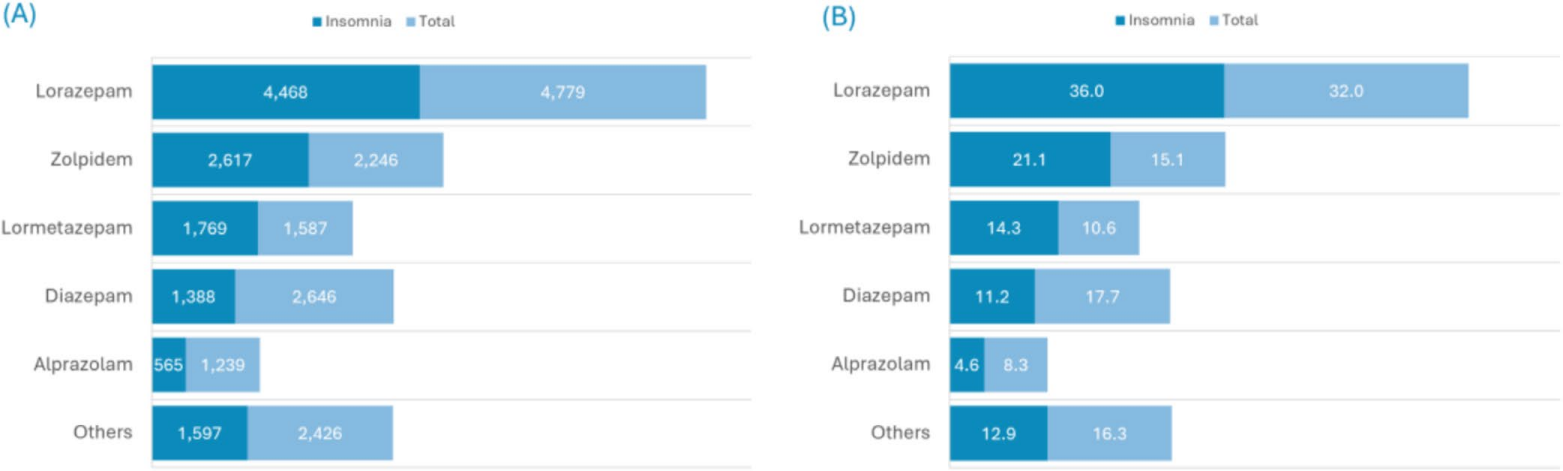
(A) Absolute and (B) relative frequency (%) of prescriptions of hypnotic‐sedatives for any use and for insomnia between 2009 and 2017 in the Sagunto Health Department (Valencia, Spain). Data relative to the total of prescriptions. *Source:* Primary care data from the Minimum Data Set (CMBD) from the Valencian Community, and the National Statistics Institute (INE).

When all hypnotic‐sedatives prescribed during the same period were taken into account, both for insomnia and for other health problems, the percentages corresponding to the different compounds changed slightly. Lorazepam remained the most widely used compound (32.0%), but diazepam now ranked second (17.7%), followed by zolpidem (15.0%) and lormetazepam (10.6%). Alprazolam rose to 8.3%. These changes in usage patterns probably reflect the other indications of these compounds in other psychological, neurological or psychiatric processes, such as anxiety and depression.

Thirty‐two percent of patients with insomnia did not receive a prescription for hypnotics at their first consultation (*n* = 7082). In 54% of cases, more hypnotics were recorded than the number of consultations (*n* = 11,926), while in 13.6% of cases, more consultations were recorded than the number of psychotropic drugs prescribed. Potential trends in the prescription of most commonly drugs for insomnia management in Spain are shown in Figure 4.

**FIGURE 4:**
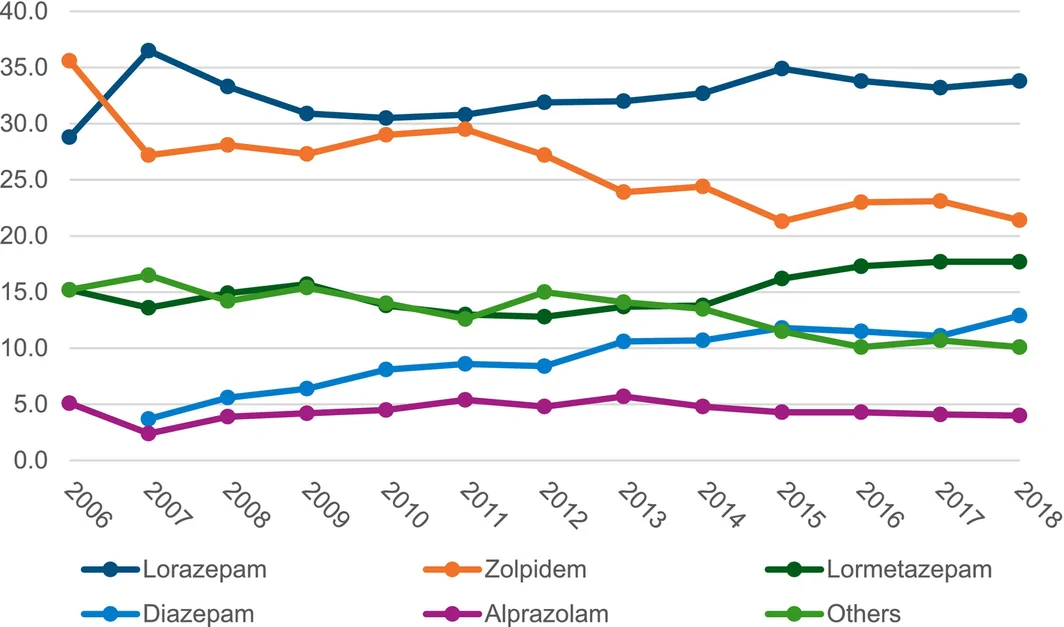
Evolution of the most common drugs for insomnia (percentage) 2006–2018.

In terms of comorbidities coded in the system, common cold (among all health conditions) and restless legs syndrome (among all sleep disorders) were the most frequently associated diseases in patients with an initial diagnosis of chronic insomnia.

## 4 Conclusions

The results show a high incidence of insomnia, which is on the rise. In the survey by de Entrambasaguas et al. (2023), with a more accurate but less accessible definition for analysis with Big Data, the prevalence of insomnia symptoms was 43.4%, chronic insomnia syndrome was 13.7% and chronic insomnia disorder was 14.0% (women 14.6%, men 13.4%; 18–34 years 11.1%, 35–54 years 11.5% and 55 years or older 17.9%). The data from our analysis are closer to the insomnia symptoms prevalence in the Entrambasaguas's survey than to their insomnia prevalence, probably in relation to the diagnosis being made in our case by PC physicians, which could be classified as ‘clinical insomnia’. This difference highlights that the excessive prescription of hypnotic drugs in PC may be related to insufficient assessment of the diagnostic criteria for insomnia disorder, and a lack of non‐pharmacological interventions being offered in the first instance, following a stepped‐care model. Our data are also in the highest range and even above the range of estimated prevalence rates reported by region for clinical insomnia in the RAND report (Hafner et al. 2023). The cumulative incidence, equivalent to prevalence, of insomnia during the study period reached 26% of the health area.

The increase in the frequency of sleep disorders is well‐known in the literature, with some independent risk factors having been described, such as age, Caucasian race, high cultural level, smoking, the presence of high blood pressure, diabetes, heart disease and a BMI ≥ 25 (Nie et al. 2024). Some authors also suggest that the observed increase may be due to overdiagnosis or the use of a very broad definition in clinical practice (Wang et al. 2023; Lopez‐Rodriguez 2018), as our study also implies.

Concerning pharmacological management, the analysis reveals a predominant use of BZDs to treat insomnia, with lorazepam being the most frequently prescribed. This contrasts with current recommendations on the use of non‐pharmacological interventions as the first line of treatment for chronic insomnia. The European guideline published in 2023 recommends CBT as the first treatment for insomnia in adults of any age, even with comorbidity, and the use of pharmacological compounds, such as BZDs up to 4 weeks, as an alternative or adjuvant treatment (Riemann et al. 2023). Unfortunately, the value of non‐pharmacological measures is underestimated by both doctors and patients (Hrehova and Mezian 2021), despite the fact that a multidisciplinary approach is crucial for the treatment of insomnia and the prevention of BZD abuse, which can lead to serious health problems.

It is important to note that in 54% of cases, there were more prescriptions for psychotropic drugs than medical consultations. This result suggests that prescriptions may be automatically renewed without proper patient assessment, leading to prolonged and unnecessary use of these drugs.

Furthermore, we found that a significant percentage of prescriptions were for indications other than insomnia, an some of which may be inappropriate or off‐label use (Barboza Zanetti et al. 2024). Many of these treatments may have been initiated as treatment for other short‐term disorders or hospital stays, and then continued due to therapeutic inertia, which tends to perpetuate into old age, when it is even more common and problematic (Wang et al. 2023). There are formal clinical trials of de‐prescribing approaches used in other countries that could be used in necessary cases (Coteur et al. 2023).

According to the Spanish Medicines Agency (AEMPS), the consumption of anxiolytics, hypnotics and sedatives has risen by more than 10 percentage points between 2010 and 2021, from 82.50 daily doses per 1000 inhabitants in 2010 to 93.04 daily doses per 1000 inhabitants in 2021 (Agencia Española del Medicamento 2025). It should be noted that 15.8% of Spaniards claim to have consumed opioid analgesics or hypnotics without a prescription at some point during the past year, according to the 2022 report published by the Ministry of Health at the beginning of 2023 (Agencia Española del Medicamento 2025; ConSalud.es 2025).

One strength of our analysis is the long observation period, the large amount of data collected, which reflects the reality of current medical practice in our primary care area in our environment. Moreover, it was carried out before the COVID‐19 pandemic, and is therefore free from its influence on consultations and prescriptions during that period. However, the study has limitations, such as variability in diagnostic coding, clinical information and drug prescription by professionals, as well as a lack of information on the severity of insomnia and other factors that may influence prescribing decisions. The current coding system for insomnia in primary care does not allow for distinguishing between the different types of insomnia, their aetiology or their chronicity. The results of this study may help to improve and refine this coding system.

On the other hand, we acknowledge that restricting the study to the Sagunto health department limits geographic generalisability. This study was part of the analysis conducted before deploying an implementation intervention and strategy that required a local context. Nevertheless, the department's mixed urban–rural composition and population size provide variability in sociodemographic characteristics that partially mitigates this limitation. Results should therefore be generalised cautiously to populations with substantially different healthcare organisations, sociodemographic profiles or urbanicity.

At the time of the study, the Sagunto Health Department lacked a multidisciplinary Sleep Unit equipped with consultation services and sleep testing facilities. This context is highly representative of most Spanish health areas, where access to specialised Sleep Units remains challenging and delayed.

In conclusion, the management of insomnia in PC in our area during the analysed period revealed issues of excessive pharmacological treatment of insomnia, especially prescription of BZDs, and a lack of follow‐up. Addressing these problems as suggested is essential for improving patient care.

## Author Contributions

**Jesús Escribá‐Alepuz:** methodology, formal analysis, writing – original draft. **Lidia Herrero‐Huertas:** methodology, writing – review and editing. **María García‐Gil:** software, writing – review and editing. **Estefanía Estévez‐López:** writing – review and editing. **Francisco Javier Puertas:** conceptualization, writing – original draft, formal analysis. **Manuel de Entrambasaguas:** writing – review and editing.

## Funding

Inmusc provided medical writing assistance throughout an unrestricted grant from Idorsia Pharmaceuticals.

## Ethics Statement

This study uses deidentified aggregated data and, as such, is not subject to approval by an Ethics Committee. Nevertheless, the principal investigator sought approval of the protocol from the Ethics Committee for Research with Drugs at Hospital de Sagunto, which was approved (Acta 2017/11).

## Conflicts of Interest

The authors declare no conflicts of interest.

## Data Availability

The data that support the findings of this study are available on request from the corresponding author. The data are not publicly available due to privacy or ethical restrictions.
